# Delayed Horner’s Syndrome Post Thyroid Carcinoma Surgery

**DOI:** 10.7759/cureus.38046

**Published:** 2023-04-24

**Authors:** Sangeetha Manoharan, Mushawiahti Mustapha, Shahrun Nizam Abdullah Suhaimi, Norshamsiah Md Din

**Affiliations:** 1 Ophthalmology, Universiti Kebangsaan Malaysia Medical Centre, Kuala Lumpur, MYS; 2 Breast and Endocrine Surgery, Universiti Kebangsaan Malaysia Medical Centre, Kuala Lumpur, MYS

**Keywords:** horner’s syndrome, complication, sympathetic, postoperative, thyroid carcinoma, delayed

## Abstract

Post-thyroidectomy Horner’s syndrome (HS) is a rare occurrence, and its probability increases when a modified radical neck dissection is performed. We present a case of a patient with papillary thyroid carcinoma who presented with Horner’s syndrome one week after the right lateral dissection of the cervical lymph nodes. She underwent a complete thyroidectomy four months prior to this surgery. Both surgeries were uneventful intraoperatively. On examination, the right eye (RE) had partial ptosis with miosis and the absence of anhidrosis. A pharmacological test with phenylephrine 1% was used to localize the interruption of the oculosympathetic pathway with postganglionic third-order neuron involvement. She was treated conservatively, and her symptoms improved over time. Horner’s syndrome is a rare and benign complication of post-thyroidectomy surgery with radical neck dissection surgery. Since it does not compromise visual acuity, the disease is constantly overlooked. However, in view of the facial disfigurement and the possibility of incomplete recovery, the patient needs to be forewarned regarding this complication.

## Introduction

Horner’s syndrome (HS) is clinically defined as ipsilateral blepharoptosis, pupillary miosis, ipsilateral enophthalmos, and facial anhidrosis, which occurs following the injury affecting the oculosympathetic pathway [1]. The occurrence of HS can be due to many causes depending on where the injury to the oculosympathetic pathway takes place. This syndrome does not affect vision; however, it can indicate the presence of an underlying health problem that may be very serious. One of the many etiologies of developing HS will be thyroidectomy surgery. Post-thyroidectomy HS is a rare occurrence with an incidence rate of 0.2%-0.27%, and its probability increases when a modified radical neck dissection is done [2-4]. Our patient developed HS only one week after surgery, which is rare since most patients become symptomatic within 2-4 days after surgery.

## Case presentation

This article was previously presented as a meeting abstract at the 11th Conjoint Ophthalmology Scientific Conference/Universiti Malaya-Asia Pacific Ophthalmic Trauma Society (UM-APOTS) Ophthalmic Trauma Meeting 2022 webinar on September 17-18, 2022. Herein, we report a 34-year-old female who presented with right eye (RE) ptosis and miosis a week after right lateral cervical lymph node dissection following total thyroidectomy. The patient was referred to us a month after surgical intervention for thyroid cancer. In April 2020, she was diagnosed to have a solitary thyroid nodule and subsequently had multiple thyroid surgeries. First, she underwent a right hemithyroidectomy in April 2020. Histopathological examination confirmed a papillary thyroid carcinoma with lympho-vascular invasion. Subsequently, she underwent a complete thyroidectomy in October 2020. Lymph nodes were not removed intraoperatively as they appeared normal. A histopathological examination of the left thyroid gland showed lymphocytic thyroiditis. During her follow-up review, enlarge right cervical lymph node was noted on palpation. An ultrasound of the neck revealed multiple enlarge right-sided lymph nodes over levels II and III (between the skull base and cricoid cartilage). An iodine-131 whole-body scan done in March 2021 showed radioactive iodine-avid right cervical lymph node metastasis. Subsequently, the right lateral dissection of the cervical lymph node encompassing regions II-IV (lymph nodes along the jugular vein from the skull base to the clavicle) was performed in April 2021. The surgery was uneventful.

**Figure 1:**
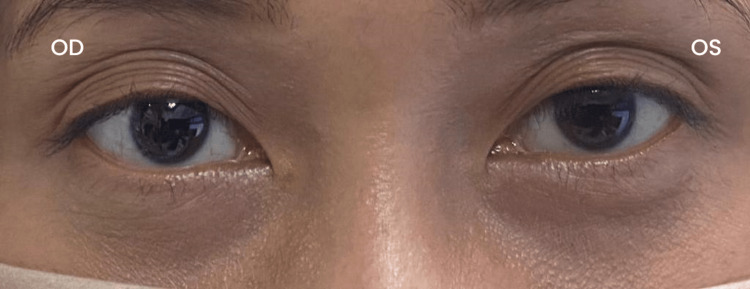
The right eye (OD) does show partial ptosis, and the left eye (OS) does not show ptosis post surgery

The patient complained of right eyelid ptosis a week after surgery. Further examination showed miosis but no anhidrosis (Figure 1). She did not complain of any blurring of vision nor any other complications, such as bleeding, wound infection, change in voice, or any symptoms suggesting hypoparathyroidism.

**Figure 2:**
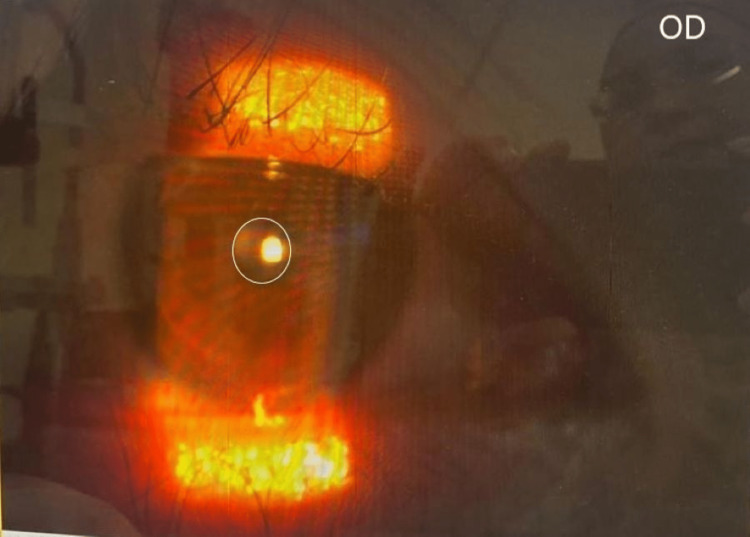
The right eye (OD) pupil is 2 mm

**Figure 3:**
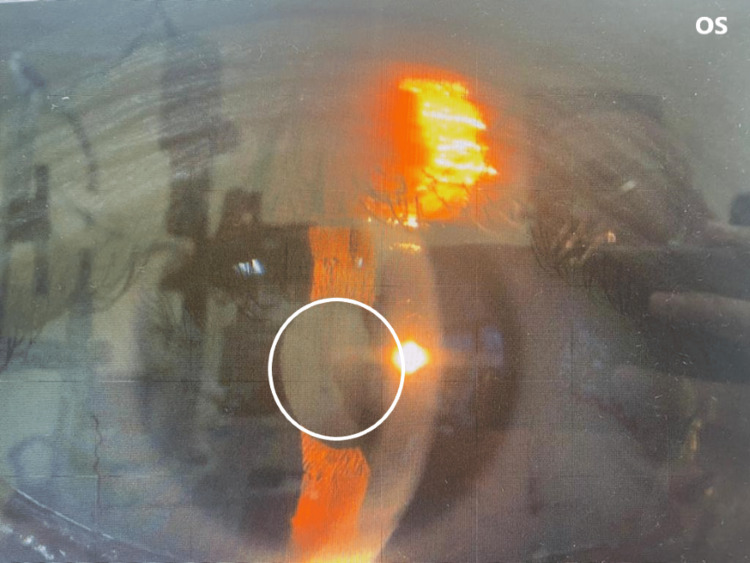
The left eye (OS) pupil is 4 mm

On examination, her best-corrected visual acuity (BCVA) was 6/7.5 in the right eye and 6/6 in the left eye (LE). There was partial ptosis of the right eyelid, which was not obscuring the visual axis. The right eye pupil was smaller at 2 mm compared to 4 mm over the left eye (Figures 2-3). There was no obvious anhidrosis and enophthalmos. The rest of the ocular examination was normal. The examination of the left eye was unremarkable. The neck showed mild skin and soft-tissue edema, without postoperative hematoma or lymphostasis. A pharmacological test with low-dose phenylephrine 1% was used to localized the interruption of the oculosympathetic pathway. Phenylephrine 1% was prepared by diluting phenylephrine hydrochloride 10% (0.1 ml) with 0.9 ml of preservative-free natural teardrops. Her pupils were measured in the same room lighting with a standard ruler one hour before and after the instillation of two drops. Post instillation of the eye drops, the RE pupil dilated, but the LE pupil did not dilate. This concludes that the patient has right-sided Horner’s syndrome with the lesion localized to the postganglionic third-order neuron. She was treated conservatively, and her symptoms improved over time.

## Discussion

The ocular cervical sympathetic chain comprises three different neurons, and any lesion along this pathway results in HS [5]. The first-order neurons originate from the hypothalamus and synapse in the spinal cord at the level of C8-T2. The second-order preganglionic pupillomotor fibers exit the spinal cord at the level of T1 and enter the cervical sympathetic chain. It then ascends through the pulmonary apex to synapse in the superior cervical ganglion, which is at the level of the bifurcation of the common carotid artery (C3-C4). Axons from the third-order neuron originating in the superior cervical ganglion travel along the carotid artery system to reach the orbit [5].

Our patient developed right eye blepharoptosis and miosis with the absence of anhidrosis. This is because axons of the third-order neuron will travel as plexus along the carotid artery into the cavernous sinus, through the superior orbital fissure, and into the orbital cavity. Sympathetic fibers destined to innervate the pupillary dilator muscle and Müller’s muscle will travel along the axons through the ciliary ganglion via the long ciliary nerves. The disruption of this pathway results in the relaxation of Müller’s muscle, resulting in ptosis, and the uninhibited parasympathetic supply results in the constriction of the pupil (miosis). Anhidrosis is usually an identifiable symptom of HS because the sudomotor fibers that supply the sweat glands also branch of from the third-order ganglion. In our patient, we postulate that the insult was distal to the superior cervical ganglion or after the branching of the sudomotor fiber to explain the absence of anhidrosis [5].

The patient developed HS after the right lateral dissection of the cervical lymph nodes of levels II-IV and not after her thyroidectomy surgery. This is because the cervical sympathetic plexus is wrapped around the carotid artery; therefore, the dissection of level III lymph nodes, mainly those found lying between the hyoid bone and the inferior aspect of the thyroid cartilage, may result in HS [6] due to the close anatomical relationship of the underlying sympathetic chain [3]. However, despite its close proximity, the development of HS in our patient was delayed (postoperatively one week). Based on the literature, we hypothesize that the sympathetic chain was retracted during the removal of the lymph node over regions II-IV [7]. Postoperative edema and inflammation will compress the sympathetic chain resulting in ischemia and the temporary loss of function of the cervical sympathetic nervous system leading to delayed presentation.

There are several other theories to explain the possible reasons for HS. This includes direct damage of the stellate ganglion, strain of the sympathetic trunk during lateral retraction, ischemia causing neural impairment, postoperative hematoma compressing the cervical sympathetic chain, and damage of the communication between the cervical sympathetic trunk and the recurrent laryngeal nerve [7]. However, this will result in a more acute presentation of Horner’s syndrome, which does not correlate with our patient.

Another focus of discussion in this case is the use of pharmacological drug to diagnose post-thyroidectomy Horner’s syndrome. Because post-thyroidectomy HS is caused by the disruption of the third-order neuron in the sympathetic chain, two drugs can be used to diagnose HS, which are phenylephrine 1% and hydroxyamphetamine 1% [8]. Deciding pre- or postganglionic neuron involvement of HS is crucial as it guides the physician in the further management of the disease.

Phenylephrine is a sympathomimetic agent and has a stimulating action on sympathetically innervated effector cells [9]. However, if an organ is deprived of its normal innervation, it will become supersensitive to the chemical mediators normally released from those nerves [9]. Phenylephrine 1% has a mild mydriatic effect on the pupillary dilator muscle. Due to the supersensitivity theory, a denervated muscle (postganglionic lesion) will dilate in comparison to the decentralized muscle (preganglionic or central lesions). This drug has a sensitivity of 81% and a specificity of 100% [8].

Hydroxyamphetamine 1% on the other hand will dilate a normal pupil as it forces the presynaptic norepinephrine into the synaptic cleft regardless of the sympathetic activation [9]. Therefore, the presence of any lesion affecting the first- and second-order neuron of the oculosympathetic pathway will still result in pupillary dilation with hydroxyamphetamine, and the damage of the third-order neuron will result in the failure of pupillary dilatation [9]. Hydroxyamphetamine 1% has been shown to have a sensitivity of 93% and specificity of 83% [8,10].

In summary, phenylephrine 1% and hydroxyamphetamine 1% are useful in localizing the lesion to the postganglionic neuron. Both agents can be used interchangeably if one pharmacological agent is unavailable [8].

It has been estimated that 70% of patients with Horner’s syndrome after thyroidectomy will have permanent damage or incomplete recovery, while the remaining 30% will completely recover between five and 15 months after surgery [7]. A trial of IV methylprednisolone in a tapering dose for 15 days has been suggested, but no improvement in HS was seen [11], and therefore, the choice of treatment for HS remains conservative.

## Conclusions

Our report shows that delayed HS post thyroidectomy surgery is a non-life-threatening and non-vision-threatening complication, which can be easily diagnosed in a clinic setting. Despite its delayed presentation, it usually resolves spontaneously especially if a careful technique is used where peripheral ligation of the inferior thyroid artery branches and meticulous manipulation of the recurrent nerve is applied; the damage to the oculosympathetic pathway can be avoided. However, in certain unfortunate cases, there is still a possibility for incomplete recovery, and in view of that, the patient should be made aware of permanent facial disfigurement prior to surgery. We also report this case to alert the head and neck surgeon that HS can have a delayed presentation and it should not be overlooked during postoperative follow-up.
